# An assessment of the Chilean National Examination of Orthopaedic Surgery

**DOI:** 10.1186/s12909-016-0604-6

**Published:** 2016-03-01

**Authors:** Julio Urrutia, Mario Orrego, Ana C. Wright, Diego Amenabar

**Affiliations:** Department of Orthopaedic Surgery, School of Medicine, Pontificia Universidad Catolica de Chile, Marcoleta 352, Santiago, Chile; Department of Orthopaedic Surgery, Universidad de Los Andes, Santiago, Chile; Center of Medical Education, School of Medicine, Pontificia Universidad Catolica de Chile, Santiago, Chile

**Keywords:** Orthopaedic surgery, Specialty board, Resident education, Examination development

## Abstract

**Background:**

The Chilean National Examination of Orthopaedic Surgery (EMNOT) has been administered since 2009. It was developed to determine whether individual residents are meeting minimal knowledge standards at the end of their training programs.

**Methods:**

We performed a retrospective evaluation of the EMNOT for all years it has been administered (2009–2015). The test was analyzed for content, taxonomy of questions asked (1: direct recall; 2: diagnosis; 3: evaluation/decision-making), residents’ performance, difficulty index and discrimination index.

**Results:**

During the years of EMNOT administration, the most frequently tested areas have been pediatric orthopaedics (22.9 %), spine (13.8 %), general orthopaedics (13.8 %) and musculoskeletal trauma (9.9 %). A significant increase in questions with images was observed, as well as a significant decrease in the percentage of Type 1 and an increase in Type 3 questions. The Difficulty Index showed a medium level of difficulty for all years the examination has been administered. The Discrimination Index showed good discrimination in 2009, fair discrimination from 2010 through 2012, and excellent discrimination from 2013 through 2015.

**Conclusion:**

The EMNOT has evolved over several years to include better quality questions, better discrimination, and a more representative distribution of questions covering the different orthopaedic sub-specialties. This examination represents an effective instrument for quality assurance of orthopaedic residency programs in Chile.

## Background

In recent decades, there has been a dramatic increase in the levels of scientific knowledge, diagnostic expertise and overall competence that orthopaedic surgery residents must master during their training. This growing amount of information creates a need for objective and standardized assessment methods to assess residents’ learning throughout their education, and especially at the end of their residency programs. In the United States of America, the Orthopaedic In-Training Examination (OITE), a national test taken annually by orthopaedic residents that evaluates knowledge of the most important subjects in orthopaedic surgery, has been assessed in several studies [1–4]. However, almost no information is available in the medical literature about similar evaluations for orthopaedic surgery residents in Latin America.

In Chile, the rising expectations of patients, combined with the country’s economic growth and its aging population, have required that more new physicians be trained in different specialties. In response to this demand, the number of orthopaedic surgery residency programs throughout the country has grown during the last decade. As a result, the number of orthopaedic surgeons graduating each year in Chile has also increased significantly. The Chilean Society of Orthopaedic Surgery (SCHOT) plays an active role in the continuing education of orthopaedic surgeons and residents in Chile, and many of the Society’s active members serve as certifying experts to ensure the quality of training programs. This is similar to the other medical societies in Latin America, which have played an active role in quality assurance for specialty training [5–7].

To determine whether individual residents are meeting minimal knowledge standards at the end of their training programs, the SCHOT developed the EMNOT, an acronym for “Examen Medico Nacional de Ortopedia y Traumatologia” (National Medical Examination in Orthopaedic Surgery). The EMNOT is a multiple-choice exam covering the areas of general orthopaedics (including basic sciences); musculoskeletal trauma; pediatric orthopaedics; spine; shoulder and elbow; hand; hip and pelvis; knee and sports medicine; foot and ankle; and musculoskeletal oncology. The EMNOT was first administered in 2009, and, to date, a total of 279 final-year residents have taken the test.

In this study, we analyzed this educational tool by assessing the examination’s distribution of questions in the different subspecialties of orthopaedics, its question taxonomy, and its difficulty and discrimination indices.

## Methods

For this study, we first obtained institutional board approval from the President and the Board of Directors of the SCHOT and its ethics committee. The information we analyzed did not include the examinees’ and institutions’ identifying data.

We retrospectively assessed the 7-year period during which the examination has been administered (2009–2015). The total number of residents taking the EMNOT (each year and during the entire period) was recorded. All questions were categorized using the taxonomic classification described by Buckwalter et al. [8]. A question was defined as Type 1 (pure knowledge) when it required the recall of facts but no interpretation; as Type 2 (diagnosis) if it required interpretation of information provided (including images); and as Type 3 (evaluation/decision-making) if the resident needed to decide on a treatment plan using the data provided. Any discrepancies regarding the categorization of any question were discussed in a joint review and resolved based on consensus.

Each question was also classified into one of ten areas: general orthopaedics (including basic sciences); musculoskeletal trauma; pediatric orthopaedics; spine; shoulder and elbow; hand; hip and pelvis; knee and sports medicine; foot and ankle; and musculoskeletal oncology. The percentage of questions from each area as a fraction of the entire EMNOT was established. Residents’ performance on the complete examination, as well as on each taxonomic type of question, was recorded.

The Difficulty Index (P) was determined as described by Crocker and Algina [9]. P refers to the percentage of correct responses to the test item; it was calculated using the formula *P* = R/T, where R is the number of correct responses and T is the total number of responses (i.e., correct + incorrect + blank responses). Levels of P were established as proposed by Backhoff et al. [10], with values < 5 % considered difficult; 5 – 25 % considered medium-hard difficulty; 26 – 75 % considered medium difficulty; 76 – 95 % considered medium-low difficulty and > 95 % considered low difficulty.

Finally, we calculated the Discrimination Index (D). D refers to the capacity of an item to discriminate between high-ability examinees and low-ability examinees. We first scored each examinee's test and rank-ordered the test scores; next, the top 50 % of students (high-ability examinees) and the bottom 50 % (low-ability examinees) were separated for analysis. D for each question is the number of examinees in the upper group who answered the item correctly minus the number of examinees in the lower group who answered the item correctly, divided by 50 % of the number of students taking the test each year. Levels of D were established as proposed by Ebel and Frisbie [11], with D values 0.00–0.20 considered poor discrimination; 0.20–0.29 considered fair discrimination; 0.30–0.39 considered good discrimination and > 0.39 considered excellent discrimination.

Statistical analysis was conducted using Statistical Program for the Social Sciences (SPSS) version 18 (SPSS, Chicago, IL). Categorical variables were expressed as percentages. Fisher’s exact test was used to analyze categorical variables. A p value less than 0.05 was considered statistically significant.

## Results

A total of 279 residents finishing their orthopaedic surgery programs have taken the EMNOT since 2009; the number of examinees has increased each year, as shown in Table 1.

**Table 1:** General description of the test from 2009 to 2015

	2009	2010	2011	2012	2013	2014	2015
Number of examinees	23	26	32	38	49	55	56
Number of questions	90	110	120	120	120	120	120
Number of questions with images	0	0	4	16	36	34	24
Difficulty Index (median)	66.0	71.6	55.8	52.7	62.4	65.9	65.2
Discrimination Index (median)	0.34	0.28	0.27	0.25	0.41	0.41	0.58

The number of questions increased from 90 in 2009 to 110 in 2010, and remained at 120 from 2011 to 2015. The number of questions containing some type of image has increased from zero questions containing images in 2009 and 2010 to a median of 34 questions containing images during the last three years (2013 – 2015), *p* < 0.01 (Table 1).

The proportion of Type 1 questions has decreased significantly (from 77.8 % in 2009 to 59.2 % in 2015). There has also been a significant increase in Type 3 questions (from 5.6 % in 2009 to 22.5 % in 2015), *p* < 0.05. Variations in the proportion of questions by taxonomy level are shown in Tables 2 and 3.

**Table 2:** Percentage of questions according to taxonomic classification

	Year							
	2009	2010	2011	2012	2013	2014	2015	Mean ± SD
Type 1	77.8	76.4	65.8	59.2	52.5	59.2	59.2	64.3 ± 9.6
Type 2	16.7	17.3	25.8	25.0	22.5	21.7	18.3	21.0 ± 3.7
Type 3	5.6	6.4	8.3	15.8	25.0	19.2	22.5	14.7 ± 8.0

*SD* standard deviation

**Table 3:** Number of total questions according to taxonomic classification

	2009	2010	2011	2012	2013	2014	2015
Type 1	70	84	79	71	63	71	71
Type 2	15	19	31	30	27	26	22
Type 3	5	7	10	19	30	23	27
Total	90	110	120	120	120	120	120

The most frequently tested areas have been pediatric orthopaedics (22.9 %), spine (13.8 %), general orthopaedics (13.8 %) and musculoskeletal trauma (9.9 %), as shown in Table 4.

**Table 4:** Percentage of questions by area

Area	Year							
	2009	2010	2011	2012	2013	2014	2015	Total
Hip/pelvis	2.2	8.2	5.8	6.7	6.5	7.7	6.7	6.3
Spine	21.1	15.5	13.3	12.5	9.8	14.5	10.0	13.8
General orthopaedics	2.2	7.3	2.5	6.7	11.4	12.0	12.5	7.8
Shoulder/elbow	8.9	4.5	11.7	10.8	9.8	4.3	9.2	8.4
Pediatric orthopaedics	28.9	22.7	24.2	19.2	20.3	22.2	22.5	22.9
Hand	14.4	10.9	10.0	7.5	6.5	6.8	7.5	9.1
Knee/Sports Medicine	10.0	10.0	9.2	9.2	9.8	7.7	8.3	9.2
Foot and Ankle	2.2	8.2	5.8	6.7	7.3	8.5	7.5	6.6
Musculoskeletal Trauma	5.6	4.5	12.5	11.7	11.4	11.1	12.5	9.9
Tumor	4.4	8.2	5.0	9.2	7.3	5.1	3.3	6.1
Total	100	100	100	100	100	100	100	100

The Difficulty Index of the test (median *P* = 65.2; range 52.7 – 71.6) showed that the test has presented medium difficulty in each of the years it has been administered. The Discrimination Index (median D = 0.34; range 0.25 – 0.58) showed good discrimination in 2009 but only fair discrimination from 2010 through 2012; D values increased to excellent discrimination from 2013 through 2015, as shown in Table 1.

## Discussion

The SCHOT has been administering the EMNOT since 2009 to assess whether residents finishing their orthopaedic surgery training programs are meeting minimal knowledge standards. Although a multiple-choice examination like the EMNOT cannot directly assess all the necessary competencies required of orthopaedic surgeons [12], it serves as a standardized evaluation tool of cognitive competencies. This is an extremely important function given that medical knowledge influences the quality of patients’ care [13, 14].

Our study shows that despite a decrease in the number of knowledge questions (Type 1) and an increase in the number of evaluation/decision-making questions (Type 3), the proportion of correct answers did not change during the administration period of the test (Table 5). Therefore, the variation in the taxonomy of questions did not affect the performance of the examinees; similar results have been described in other studies evaluating orthopaedic tests [1]. Accordingly, no significant differences in the Difficulty Index of the test were observed throughout its years of administration. This is important because Type 3 questions, as opposed to questions that require only the recall of data, better evaluate the competencies expected from a specialist.

**Table 5:** Percentage of correct answers according to taxonomic classification

	Year						
	2009	2010	2011	2012	2013	2014	2015
Type 1	63.1	70.5	65.0	60.3	58.3	59.7	62.4
Type 2	63.2	77.5	65.5	60.5	60.8	61.0	59.9
Type 3	61.5	68.1	57.1	65.6	68.1	61.1	60.8

The EMNOT migrated from a paper-based exam to a computer-based exam in 2012. This change facilitated the inclusion of images, which resulted in an increased percentage of questions involving figures. There were no images in 2009 and 2010 and the test had a median of 34 questions with images from 2013 to 2015. This also helped with the development of type 2 and type 3 questions: the inclusion of radiographs, patient photos, and graphs has resulted in more intricate questions that require a more complex approach, requiring the examinee to provide diagnosis and treatment decisions.

This examination was developed as a comprehensive examination testing core knowledge in all areas of orthopaedic surgery. In this analysis, we found that not all sub-specialties were proportionately represented by the test; pediatric orthopaedics and spine surgery were the most represented sub-specialties. Similar results were also found by Papp et al. in their report on the OITE exam [2]. In our results, pediatric questions represented 22.9 % of all questions; this proportion reflects the time allocated to this sub-specialty, which represents between 18 and 24 % of an orthopaedic residency, depending on the program. The high ratio of spine questions may be partly explained by the fact that all pediatric spinal deformity questions were labelled as spine questions; nevertheless, this over-representation has been corrected during the most recent years of the examination, as shown in Table 4.

The test has evolved throughout the years, with an increase in the total number of questions, an increase in Type 2 and Type 3 questions, and a more homogeneous representation of the different sub-specialties. The Difficulty Index shows that the exam has had a medium difficulty level throughout all years of administration; the Discrimination Index shows that the test has increased its discrimination capacity in the last three years. While these results are encouraging, the EMNOT still faces the limitation that, as a multiple-choice examination, it only evaluates knowledge, diagnostic skills, and evaluation/decision-making skills. The assessment of surgical skills and attitudes of examinees still remains a challenge [15, 16].

Although beyond the scope of this article, the results of these exams have also helped to identify learning needs for the development of a more relevant continuing education program. Moreover, yearly reports are given to Residency Program Directors in Chile to aid in curriculum development.

In a recent review, Gurgacz et al. postulated that a socially useful credentialing process in surgical specialties should adhere to three main conditions: (a) only one institution should be responsible for the credentialing process, (b) best-practice standards for design, implementation, and monitoring of the examination should be used and (c) the organization should have a strong quality-improvement culture [17]. The SCHOT develops the EMNOT with the help of experts in orthopaedic surgery and medical education. This exam has been evaluated yearly to improve its standards and to establish a certification process. We believe that future evaluations of the EMNOT should include national and international appraisals to ensure an external impartial verification of its quality level. The examination should also incorporate methods for the assessment of surgical skills and attitudes among graduating orthopaedic residents.

## Conclusion

The EMNOT has evolved over several years to include higher quality questions, better discrimination, and a more representative distribution of questions covering the different orthopaedic sub-specialties. Thus, this examination developed and administered by the SCHOT represents an effective instrument for quality assurance of orthopaedic residency programs in Chile.
